# Free Care Is Not Enough: Barriers to Attending Free Clinic Visits in a Sample of Uninsured Individuals with Diabetes

**DOI:** 10.4236/ojn.2014.413097

**Published:** 2014-12-01

**Authors:** Jennifer A. Mallow, Laurie A. Theeke, Emily R. Barnes, Tara Whetsel, Brian K. Mallow

**Affiliations:** 1 School of Nursing, West Virginia University, Morgantown, West Virginia, USA; 2 Sovern Run, LLC, Albright, West Virginia, USA

**Keywords:** Diabetes, Rural Health, Poor, Uninsured, Free Clinic, Chronic Conditions, Health Disparities

## Abstract

Free care does not always lead to improved outcomes. Attendance at free clinic appointments is unpredictable. Understanding barriers to care could identify innovative interventions. The purpose of this study was to examine patient characteristics, biophysical outcomes, and health care utilization in uninsured persons with diabetes at a free clinic. A sample of 3139 patients with at least one chronic condition was identified and comparisons were made between two groups: those who attended all scheduled appointments and those who did not. Geographic distance to clinic and multiple chronic conditions were identified as barriers to attendance. After one year, missing more than one visit had a positive correlation with increased weight, A1C, and lipids. Additionally, patients who missed visits had higher blood pressure, depression scores, and numbers of medications. Future research should further enhance understanding of barriers to care, build knowledge of how social and behavioral determinants contribute to negative outcomes in the context of rurality. Innovative methods to deliver more frequent and intensive interventions will not be successful if they are not accessible to patients.

## 1. Introduction

Attendance at free clinic appointments is unpredictable, and cancellation rates are high [1], particularly in populations that experience significant disparity and multiple poorly controlled chronic conditions. The treatment of chronic conditions now accounts for over 84% of current national healthcare expenditure [2]. According to the Center for Disease Control, chronic conditions, such as diabetes, heart disease, and stroke, are among the most prevalent, costly, and preventable of all health problems [3]. Additionally, chronic conditions are the leading causes of death and disability in America. Almost half of the American population is living with at least one chronic condition and rates are expected to continue to increase [4]. Furthermore, healthcare disparities exist related to chronic condition care and outcomes.

Healthcare disparities can be defined as decreased access to or availability of healthcare services. Health disparity refers to the disproportionate rates of disease and disability in a socioeconomically poor or geographically isolated population [5]. Patients in rural areas are more likely than patients in urban areas to be uninsured or underinsured and to report deferred care due to cost. Further, they are less likely to have recommended preventative health screenings than those in urban populations [6]. The Appalachian region, which consists of 13 Eastern states, is recognized as an area of both healthcare and health disparity.

The proportion of residents with chronic conditions and poor health outcomes of chronic conditions is higher in Appalachia when compared with those in the rest of the nation [7]. West Virginia is in the only state that is entirely in Appalachia. West Virginia experiences multiple physical, social, and behavioral determinants of health. West Virginia ranks 48th in the nation for lowest number of citizens with a high school graduation, highest incidence of infectious disease, highest prevalence of low birth-weight infants, highest underinsured population and low availability of primary care providers. Considerable resources have been dedicated to diminish disparity in the Appalachian region but long-term successes have not been documented.

Federally Qualified Health Centers (FQHC) and free clinics were developed as potential solutions to healthcare disparities in West Virginia. These healthcare safety nets were originally meant to enhance access to care by providing comprehensive health services to medically underserved populations with the secondary goal of reducing patient load on hospital emergency rooms. All persons, regardless of ability to pay, are able to receive care through FQHCs or free clinics. Free clinics and FQHCs provide over 19.6% of the population with free or reduced cost care in West Virginia [8]. Though a system of free clinics and FQHCs exists, 17.7% of the population in West Virginia reported that they couldn't seek medical care due to cost, which is higher than the national average of 14.6% [9].

There are hidden costs associated with healthcare attendance that may be contributing to poor attendance or low care seeking rates. These hidden costs include inability to quickly access care due to distance, lack of an interstate transportation system, lack of a personal automobile, lack of well-developed public transportation systems, and overall cost of transportation [10]. Personal economic conditions in the state may make it too difficult for people to seek care, even if insured. In addition, the overall low number of healthcare providers and facilities continue to play a prominent role in poor chronic condition outcomes, specifically in Appalachia [7].

Detrimental biophysical outcomes and multifaceted patient characteristics of low-income, uninsured Appalachian population make targeting specific interventions complex. Barriers affect a person's ability and willingness to obtain needed care. Understanding the true barriers to care may enhance knowledge by identifying new target areas for innovative patient centered healthcare system interventions. Little is still known about the characteristics of patients who do not attend free visits and how the lack of attendance truly impacts multiple chronic conditions. Reduced fee or free care may not be enough to improve outcomes in this population. Studies that include barriers other than cost of office visits could result in new knowledge that leads to improved healthcare, and thus, improved chronic condition outcomes. Diabetes was chosen as the chronic condition for the study and presented in this paper. Diabetes was chosen based on our findings in a previous intervention study at the clinical site during which it was discovered that less than 50% of persons with diabetes were attending visits in a one year time frame, making it necessary to comprehensively identify or understand barriers to attendance.

## 2. Objectives

The overall purpose of this study was to examine patient characteristics, biophysical outcomes, and health care utilization in uninsured and underinsured persons with diabetes at a free clinic in West Virginia.

1. To describe the characteristics and biophysical outcomes of low-income, uninsured persons with diabetes in Appalachia.
2. To compare characteristics and biophysical outcomes of care between two groups; low-income uninsured persons with diabetes who attend their scheduled health care visits and those who do not attend their scheduled health care visits.
3. To explore the relationship between attendance at clinic, patient characteristics, and biophysical outcomes of low-income, uninsured persons with diabetes.

## 3. Methods

### 3.1. Study Design

This study is a retrospective observational cohort design using existing electronic health record data. Longitudinal data was extracted from Electronic Health Records based on the study aims, research questions, and inclusion/exclusion criteria. The results are a report based on the primary analysis of this existing data.

### 3.2. Setting

This study obtained existing medical record data from Milan Puskar Health Right, a primary care clinic that provides health care at no cost to uninsured residents of North Central West Virginia.

### 3.3. Data Extraction and Human Subjects Approval

Using data extraction technology, data from the electronic medical records, pharmacy records and front desk scheduling system were obtained and merged into a de-identified analyzable database. Data for persons who were greater than 18 years of age, diagnosed with diabetes, and uninsured or received care at a free clinic were included. Data for persons who did not have two scheduled visits within one year were excluded because lack of scheduled visits would limit ability to meet the study aim of completing one year comparisons. This study was approved by the Institutional Review Board of West Virginia University (Tracking #: 1403233403).

### 3.4. Sample

A convenience sample of all persons with diabetes who received care from May 2008, when electronic medical records were initiated, until December 2012, when the clinic changed Electronic Medical Record (EMR) systems, was identified. Data was extracted from the EMR, free pharmacy records and front desk scheduling system by the Database Administrator (BKM) and transitioned into a Structured Query Language (SQL) database. Then, based on the research questions for this study, data was imported into Statistical Package of Social Sciences (SPSS), version 18 for analysis. The database for analysis held no patient identifiers and no information was able to be linked back to patients.

Initially, 7842 patient records were extracted. Eliminating patients that did not have more than one visit scheduled within the study time frame resulted in a decreased potential sample size of 6071 records. Finally, after limiting to only records of patients with diabetes by using diagnosis codes, medications and diagnostic criteria of diabetes, the final study sample was 3139 records of patients with diabetes.

## 4. Study Variables

### 

#### Scheduled appointments

A scheduled medical appointment was defined as any visit that the patient scheduled with the clinic. This included various types of appointments such as medical visits, mental health treatment or counseling meetings, or education sessions. Using the front desk scheduling system, we were able to separate visits that patients scheduled, canceled, or did not attend. Visits that were canceled or changed by the healthcare provider were not counted as a non-attended visit. Number of visits was coded as a numerical count; 1 visit attended = 1, and so forth. Appointments were counted for a one-year time frame.

#### Patient characteristics

The patient characteristics collected in this study included age, gender, ethnicity, marital status, education level, distance in miles and minutes from residence to clinic, depression score, types of medication, numbers of medication, types of co-morbidities and number of co-morbidities. Patient characteristic variables were collected only at baseline (first patient visit attended at the clinic within the study time frame).

#### Age

Extracted as age in years based on the age of patient at the time of the first visit within the time frame for the study. It was recorded as a continuous variable.

#### Gender

Extracted and coded as a dichotomous variable, either male or female, and coded as 0 = male and 1 = female.

#### Ethnicity

Routinely recorded on establishment of care at the clinic site as a self-reported variable. Ethnicity was coded as a categorical variable with the following categories: White, African-American, Asian, Hispanic, Native American, and other.

#### Marital status

Routinely recorded on the initial visit to the clinic and reassessed every year by patient self-report. The most recently recorded marital status was extracted and coded as a categorical variable with the following categories: single, married, divorced, separated, widowed, and significant other.

#### Education

Routinely recorded in the chart upon initial visit to the clinic. Education was extracted and coded as a categorical variable with the following categories: less than high school, graduated high school, some college, college graduate, and GED.

#### Distance to clinic and minutes to clinic

Calculated by using the address listed in the EMR and used Google's Application Programming Interface. Miles and minutes from residence to clinic were coded as continuous variables.

#### Depressive symptoms

Routinely collected at the initial visit to the clinic using The Center for Epidemiologic Studies Depression Scale (CES-D). A score of less than 15 indicates no or few depressive symptoms and behaviors during the past week. A score of 15 - 21 indicates mild to moderate depressive symptoms, while a score of over 21 indicates significant experience of depressive symptoms and the possibility of major depression. Depressive symptom scores were extracted and recorded as both a continuous variable and as a categorical variable based on number of symptoms [11].

#### Name of medication

Extracted and coded as categorical variables. There were a total of 100 categories and the category name was based on medication name.

#### Types of co-morbidities

Extracted and coded as categorical variables. There were a total of 25 categories coded as: 1 = presence of condition and 0 = no presence of condition.

#### Number of medications

Extracted and coded as a continuous number variable that represented the total count of medications documented as being currently taken by the patient.

#### Number of co-morbidities

Extracted co-morbidities were entered categorically and then a calculated variable was established to represent the total number of co-morbidities as a continuous variable.

#### Biophysical outcome of care

An outcome is a measure of a patient's clinical and functional status after an intervention has been applied [12]. Biophysics refers to the process of assigning an objective measurement to a bodily process. For the purposes of this study, a biophysical outcome of care was defined as the measurable result of care collected over a specific time frame. This study extracted the common biophysical outcomes measured in diabetes which included; *body weight,* BMI, Glycosylated Hemoglobin (A1C), blood glucose, serum creatinine, serum lipids, urine microalbumin, and blood pressure from the EMR. Biophysical outcomes of care were collected at baseline and then at a one year time point after the baseline attended appointment.

#### Body weight

Routinely measured and recorded in pounds (lbs) at the beginning of each office visit in the clinic. This number was extracted and coded into the SPSS dataset as a continuous numerical variable.

#### Height

Routinely recorded in each medical record at the initial clinical visit as height in inches. This number was extracted and coded into the SPSS dataset as a continuous numerical variable.

#### Body mass index

Calculated with the following formula: weight (lb)/[height (in)]2 × 703.

#### Percentage of glycosylated hemoglobin

Extracted and collected as a continuous variable using laboratory values that are transcribed into the EMR by clinic staff.

#### Blood glucose

Extracted and collected as a continuous variable. The clinic uses multiple means to obtain blood glucose, patient self-report via clinic provided plasma referenced, use of the same meters in the clinic, and laboratory evaluation. The EMR does not differentiate between types of measurement or between fasting and random blood glucose measurements.

#### Serum creatinine

Extracted as a continuous variable using laboratory values that were transcribed into the EMR by clinic staff.

#### Serum lipids

Extracted as a continuous variable using laboratory values that were transcribed into the EMR by clinic staff.

#### Urine microalbumin

Extracted and collected as a continuous variable using laboratory values that are transcribed into the EMR by clinic staff.

#### Blood pressure

Extracted from records after it was obtained by staff who routinely recorded the blood pressure values in the chart as systolic over diastolic.

### Data Analysis

Prior to descriptive or comparative analysis, the data were cleaned by running exploratory frequencies and descriptors. The results of the frequencies were evaluated, looking for outliers, impossible values, and patterns of missing data. Any variable item that had missing data such that it decreased power or missing data with any identifiable pattern was not analyzed.

To answer research objective 1, descriptive statistics were used. The categorical variables gender, ethnicity, marital status, education, and type of co-morbidities were analyzed using frequencies. The continuous variables age, duration of diabetes, number of co-morbidities, and miles from the clinic were analyzed using mean, median and standard deviation. The continuous variables body weight, BMI, blood glucose, A1C, creatinine, lipids, blood pressure, and microalbumin were analyzed using mean, median and standard deviation.

To answer research objective 2, the total sample size of 3139 was divided into two independent groups, those who attended all scheduled medical appointments and those who did not. Initially, the groups were compared on all variables including characteristics for significant group differences. To analyze the differences in characteristics, chi-square testing was used to assess for significant differences in the categorical variables of gender, ethnicity, marital status, education, and type of co-morbidities. Independent t-tests were used to compare means for age, duration of diabetes mellitus, miles from clinic, and number of co-morbidities. Additionally, distance traveled to clinic represented a broad range of 0.01 - 132 with a mean of 21 miles (SD 9.4). In order to complete comparisons based on true burden of travel, the research team divided this variable into a categorical variable of less than 30 miles (Mean + 1 standard deviation from the mean) and 30 miles and greater. Chi-square tests were used to look for differences in attendance based on these two categories. Finally, to assess differences in biophysical outcomes based on attendance, independent t-tests were used to compare means for body weight, BMI, A1C, blood glucose, serum creatinine, serum lipids, urine microalbumin, systolic blood pressure, and diastolic blood pressure.

To answer research objective 3, correlations were performed to examine the relationships among the number of missed visits in a free clinic over one year, body weight, BMI, A1C, blood glucose, serum creatinine, serum lipids, urine microalbumin, systolic blood pressure, and diastolic blood pressure.

## 5. Results

The results for research question 1 revealed that the sample, shown in Table 1, is mostly female, married, white, and high school educated or less. The mean age of the subjects in the study was 48 (SD 10.8, Range 21 - 64) years old. The mean number of co-morbidities was 5 (SD 1.3, Range 0 - 11) and the mean distance from the patient's home to the clinic was 21 miles (SD 9.4, Range 0.01 - 132). Table 2 summarizes the co-morbidities, emphasizing the prevalence of multiple chronic conditions in this population.

The results for research question 2 demonstrated differences based on clinic attendance. Geographic distance to clinic and multiple chronic conditions were identified as barriers to attendance. Patients who traveled 30 miles or more [X2 (3, n = 367) = 9.230, p = 0.02], and those with more than one chronic condition were more likely to miss appointments [F (3, 3131) = 430.9, p < 0.01]. There were no significant differences in the other characteristic variables of age, gender, ethnicity, marital status, education level and attendance at clinic visits. Patients who missed visits had higher blood pressure [F (3, 2493) = 1.2, p < 0.01), depression scores [F (3, 3131) = 67.4, p < 0.01] and numbers of medications [F (3, 3131) = 752.4, p < 0.01]. There were no significant differences in the other biophysical variables of creatinine or urine microalbumin based on clinic attendance.

Results for research question 3 indicated that significant relationships existed among attendance, characteristics, and biophysical measures. Missing more than one visit had a positive correlation with increased weight (r = 0.1, n = 2476, p < 0.01), A1C (r = 0.1, n = 911, p = 0.04) and lipids (r = 0.1, n = 659, p = 0.003). There were no significant relationships identified between attendance rates and baseline characteristics.

## 6. Discussion

The young mean age and multiple co-morbid conditions of this population may have contributed to missed visits. Age of patients has been associated with self-management activities such as attendance at healthcare visits. Some studies of adherence have found that age of the patient was associated with outcomes of care. Younger adult patients, less than 60 years old, have been reported to be less likely to attend education programs and multiple healthcare visits when compared to older adult patients [13]. Older adults have been shown to practice better self-management than younger adults. In addition, younger individuals were less likely to meet A1C and LDL goals [14]. Patients with a greater overall number of co-morbidities placed lower priority on each disease and had poorer self-management skills, including seeking care [15] [16].

The most common co-morbidities in this group of diabetes patients included other conditions related to metabolic syndrome, obesity, depression and pain. Obesity can lead to insulin resistance which leads to higher levels of blood glucose. The resulting increase in blood glucose can cause adverse health effects [17]. Depression and pain may have also contributed to lack of attendance. Patients with depression are more likely to experience complications of diabetes, have worse glycemic control, and be less adherent to self-care behaviors such as attendance at clinic visits than patients who are not depressed [18]. Pain has been found to limit a person's ability to perform self-management behaviors [19].

Perhaps in this population, the perceived need for healthcare is less than the actual burden to obtain care. Perceived threat of chronic illness has been identified as being significant predictors of adherence [20]. One identified burden in this population is the difference in characteristics between those who attended visits and those who did not was the distance to clinic. Those who live far distances from the clinic miss appointments. Improving access to care is best accomplished by focusing on contextual as well as individual determinates [21]. Even though care at this clinic is free, the hidden costs and burdens associated with attendance at healthcare visits remain poorly understood and require further study. This study did not look at the characteristics such as culture, social support, beliefs and genetics that may play a role in seeking care [21]. Additionally, little is known about what individual patients experience as unique barriers in the context of Appalachian culture.

### 6.1. Limitations

This retrospective data analysis included a convenience sample of persons with diabetes who received care from May 2008 until December 2012 using data extracted from the EMR. The generalizability of results is limited to the specific population of the study or to populations with similar socio-demographics or determinants of health. Additionally, only available data could be analyzed. The patient registry that was kept by the free clinic is populated by a documented diagnosis of diabetes. Upon initial data analysis, only 710 were identified as having diabetes. Using the list of diabetes medications and diagnostic criteria for diabetes (A1C greater than or equal to 6.5%) an additional 3233 cases of diabetes were identified. To account for information bias, cases having a normal A1C (less than 6.5%), only taking metformin, and no existing diagnosis of diabetes were excluded from analysis (n = 94) because of the potential diagnosis being Polycystic Ovarian Syndrome.

### 6.2. Future Research & Clinical Implications

This study highlights that access to affordable or free care is not enough to insure attendance at clinic visits. Hence, understanding barriers to obtaining care that are not linked to insurance status remains an issue. Future research should aim to further enhance understanding of individual patient perceptions of their unique barriers to care and consideration of patients' preferences on how to address their individual barriers. Understanding unique barriers and patient preferences will likely lead to the development of multiple patient centered interventions for increasing access to care.

Building knowledge of how social and behavioral determinants contribute to negative outcomes in the context of rurality is also a research priority. This study identified distance to clinic and multiple chronic conditions as barriers to obtaining care. In the current system of rural health care, the burden is placed on patients and families with very few resources to obtain necessary care. Patients may perceive burden of obtaining care, and this may outweigh the potential perceived benefits of obtaining care, particularly if patients have a poor understanding of longer term benefits of well-controlled chronic conditions. Obviously, treating complex behavioral chronic conditions such as depression in addition to physical condition such as pain and metabolic syndrome are needed in this population. However, interventions need to be culturally specific to demonstrate effectiveness. This study supports ongoing research and implementation of a substantive departure from the status quo. Namely, the approach listening to the unique needs of individual patients and developing multiple research and intervention strategies based on patient-centered specific needs and evaluating patient identified outcomes.

## 7. Conclusion

This evidence supports the need for enhanced access to healthcare for rural persons who experience multiple chronic conditions. Innovative methods to deliver more frequent and intensive interventions will not be successful if they are not accessible to patients. Future research should be multi-method and patient-centered and aim to further enhance understanding of barriers to care, build knowledge of how social and behavioral determinants contribute to negative outcomes in the context of rurality, and evaluate the impact of innovative interventions on outcomes.

## Figures and Tables

**Table 1:** Socio-demographic.

Socio-demographic	n	%
Gender		

Female	2320	73.9
Male	819	26.1

Marital Status		

Married	1585	95.5
Single	1554	49.5

Ethnicity		

White	2998	95.5
African American	113	3.6
Hispanic	28	0.9

Education Level		

Garduated High School	1243	39.6
Less than High School	763	24.3
Some College	565	18.0
GED	424	13.5
College Graduate	22	0.7

**Table 2:** Co-Morbid conditions.

Co-Morbid Conditio	n	%
Hypertention	2493	79.4
Obesity	2066	65.8
Hyperlipidemia	1811	57.7
Depression	1102	35.1
Heart disease	509	16.2
Kidney Disease	367	11.7
Pain	452	14.4
